# SENP3 mediates deSUMOylation of SIX1 to promote prostate cancer proliferation and migration

**DOI:** 10.1186/s11658-024-00665-8

**Published:** 2024-12-02

**Authors:** Zhenlong Shao, Shutong Liu, Wenshuang Sun, Xuefen Zhuang, Shusha Yin, Ji Cheng, Xiaohong Xia, Yuning Liao, Jinbao Liu, Hongbiao Huang

**Affiliations:** 1 Guangzhou Institute of Cancer Research, The Affiliated Cancer Hospital, Guangzhou Medical University, Guangzhou, 510095 China; 2 Guangzhou Municipal and Guangdong Provincial Key Laboratory of Protein Modification and Degradation, School of Basic Medical Sciences, Guangzhou Medical University, Guangzhou, 511436 Guangdong China

**Keywords:** SUMOylation, SENP3, SIX1, Prostate cancer, Tumor progression

## Abstract

**Background:**

Sentrin/SUMO-specific protease 3 (SENP3) is essential to regulate protein stability and function in normal and cancer cells. Nevertheless, its role and action mechanisms in prostate cancer (PCa) remain elusive. Thus, clarification of SENP3’s involvement and the SUMOylation process in PCa is pivotal for discovering potential targets and understanding SUMOylation dynamics.

**Methods:**

Cell viability, EdU staining, live cell imaging, and cell cycle assays were used to determine proliferation of PCa cells. Transwell and wound-healing assays were used to detect migration of PCa cells. The interaction between SENP3 and SIX1 was determined by co-immunoprecipitation, western blotting, and immunofluorescence assays. Xenograft models established on NOD-SCID mice were used to evaluate in vivo effects post SENP3 knockdown. Immunohistochemistry was performed to investigate the expression of SENP3 in PCa tissues.

**Results:**

This study found that SENP3 is highly expressed in PCa cell lines and tissues from PCa patients. Overexpressed SENP3 is associated with metastatic malignancy in PCa. Various in vivo and in vitro experiments confirmed that SENP3 promotes the proliferation and migration of PCa. In addition, SENP3 interacts with the SD domain of SIX1 and mediates its deSUMOylation and protein stability. Lys154 (K154) is required for the SUMOylation of SIX1. More importantly, SENP3 promotes the malignancy of PCa through the regulation of SIX1.

**Conclusions:**

We unravel the significant role of SENP3 in regulating protein stability of SIX1 and progression of PCa, which may deepen our understanding of the SUMOylation modification and provide a promising target for management of metastatic PCa.

**Supplementary Information:**

The online version contains supplementary material available at 10.1186/s11658-024-00665-8.

## Background

Prostate cancer (PCa) is a severe health burden and the second most common cancer in males worldwide, with 1,467,854 new cases diagnosed in 2022 [1]. Early screening strategies and local treatment of PCa can reduce mortality rates, but the insidious development often leads to late-stage diagnosis or rapid progression to metastatic disease from the primary lesion. Androgen receptor-targeted therapy has largely improved the prognosis and survival rates of patients with PCa, approximately 30–40% of patients still do not respond to this targeted treatment [2–4]. Meanwhile, patients with metastatic PCa show minimal clinical benefits from androgen pathway inhibition therapy, and the disease may even worsen. Therefore, there is an urgent need to develop alternative therapies that may replace or supply the AR signaling for the treatment of PCa.

Sine oculis homeobox homolog 1 (SIX1) is a critical transcription factor that promotes growth in organs during embryonic development, while it is silenced after embryogenesis [5]. It has been found that SIX1 signaling is reactivated in various cancers and can regulate cell proliferation and invasion by modulating the cell cycle and epithelial-mesenchymal transition [6–9]. Our previous studies have shown that the protein stability of SIX1 plays a critical role in the proliferation of PCa, suggesting that SIX1 may be a promising therapeutic target [10]. Ubiquitin-specific peptidase 1 (USP1) and the mitochondrial chaperone glucose regulatory protein 75 (GRP75) have been found to stabilize SIX1, but other posttranslational modifications of SIX1 remain unclear, posing a significant challenge to the development of targeted therapies. Therefore, further elucidation of SIX1 modification is of great importance for devising novel therapeutic strategies.

SUMOylation is a crucial reversible posttranslational modification (PTM) [11]. In essence, small ubiquitin-like modifier (SUMO) is conjugated to substrates through an enzyme-catalyzed reaction by SUMO-specific activating (E1), conjugating (E2), and ligating (E3) enzymes, similar to ubiquitination. Conversely, the deSUMOylation is regulated by deSUMOylating proteases [12, 13]. So far, three families of SUMO proteases have been identified: ubiquitin-specific protease-like 1 (USPL1) [14], deSUMOylating isopeptidase 1 and 2 (DeSI1 and DeSI2) [15], and SENPs, which are the major deSUMOylating enzymes in mammals [16]. SUMOylation is crucial for gene expression, genomic integrity, and cell cycle progression. Moreover, SUMOylation is significantly upregulated in many types of cancer [17]. These findings may indicate that cancer cells require enhanced SUMOylation to meet their diverse needs [18]. Research into SUMOylation modifications has the potential to aid in the development of therapeutic strategies for oncological treatments.

SENPs include six deSUMOylases (SENP1, SENP2, SENP3, SENP4, SENP5, SENP7) [16]. Among them, SENP3 is a key protease that can de-conjugate SUMO2/3 from modified substrates. Studies have reported that increased expression levels of SENP3 in a variety of diseases and tumors [19–22]. In the current study, we observed a significant upregulation of SENP3 in metastatic PCa. Mechanistically, we uncovered that SENP3 interacts with SIX1 and regulates its deSUMOylation, thereby enhancing its protein stabilization to promote the proliferation and migration of PCa cells. Thus, targeting the SENP3-SIX1 axis may represent a novel strategy for cancer management.

## Methods

### Agents

RPMI-1640, DMEM/F12, and Dulbecco’s modified Eagle medium (DMEM) were source from Gibco (Thermo Fisher Scientific, Waltham, MA). Fetal bovine serum (FBS) was obtained from VivaCell Biosciences (Shanghai, China). Inhibitors, including MG132 (#2619), bortezomib (#S1013), and cycloheximide (#7418), were purchased from Selleckchem (Houston, TX). *N*‐ethylmaleimide (#T3088) was purchased from TargetMol (Wellesley Hills, MA).

The following antibodies, including SENP3 (#5591), SIX1 (#16,960), SUMO2/3 (#4971), p-Rb (#8516), Rb (#9313), cyclin D1 (#55,506), E-cadherin (#8834), N-cadherin (#13,116), GAPDH (#5174), FLAG(DYKDDDK)-tag (#14,793), and HA-tag (#3724, #2367), were purchased from Cell Signaling Technology (Beverly, MA); secondary antibodies, goat anti-mouse IgG H and L (Alexa Fluor® 594) (ab150120), and goat anti-rabbit IgG H and L (Alexa Fluor® 488) (ab150077) were sourced from Abcam (Boston, MA).

### Cell culture

The following human cell lines were all purchased from the American Type Culture Collection (Manassas, VA): embryonic kidney HEK293T cells, normal prostate cell lines RWPE-1 and WPMY-1, AR-positive PCa cell lines LNCaP, 22Rv1, and C4-2, and AR-negative PCa cell lines PC3 and DU145. The identities of the cell lines were confirmed using STR Authentication, and standard mycoplasma checks confirmed no contamination. As previously described [23, 24], cell lines were grown in an incubator maintained at 37 °C temperature and 5% CO_2_. In short, the 22Rv1, LNCaP, and C4-2 cell lines were incubated in RPMI-1640 medium supplemented with 10% FBS. HEK293T, RWPE-1, and WPMY-1 were incubated in DMEM supplemented with 10% FBS. DU145 and PC3 cells were incubated in DMEM/F12 medium supplemented with 10% FBS.

### Plasmids and small interfering RNAs (siRNAs)/short hairpin RNAs (shRNAs) for transfection

FLAG-tagged full-length human SENP3 (Gene ID: 26,168) and inactive mutant SENP3-C532A were synthesized with pENTER by Kidanbio (Guangzhou, China). Full-length and point mutants human SIX1 with HA-tagged (Gene ID: 6495) were synthesized with pcDNA3.1( +) by IGEbio (Guangzhou, China). Two HA-tagged truncated mutants of SIX1 plasmid (pEnter) were purchased from VigeneBio (Shandong, China). As previously reported [25], a cocktail consisting of RPMI Opti-MEM (Gibco), Lipofectamine 3000 reagent (Invitrogen), and plasmids, added sequentially, were incubated for 15 min. The cocktail was then added to the cells in dishes or plates for 48 h for further verification and analysis.

For siRNA transfection, as previously reported [23], RPMI Opti-MEM and Lipofectamine RNAiMax (Invitrogen) were mixed with siRNAs targeting SENP3 or SIX1 and incubated for 15 min. The cocktail was then added to the cells in dishes or plates for 48 h for further verification and analysis. The sequences are as follows:

human si-SENP3-1: 5′- CCAGCATCCTCATCAGCAA -3′;

human si-SENP3-2: 5′- GCAGGACATGCCCAAACTT -3′.

human si-SIX1-1: 5′- CCAACTCTCTCCTCTGGAA -3′;

human si-SIX1-2: 5′- GCCAGGAGCTCAAACTATT-3′.

For lentivirus shRNA transfection, two pair of RNAs targeting human SENP3 were synthesized using pSH-U6-gRNA-CMV-SpCas9-P2A-puro by Vigene Biosciences (Shandong, China). After incubated with 5 μg/ml polybrene (#sc-134220, Santa Cruz) for 15 min, two independent lentiviruses were added to PCa cell culture plates at a multiplicity of infection (MOI) of ten. After transfection for 48 h, non-transfected cells would be eliminated through puromycin (#S7417, Selleck-chem) screening at a concentration of 2 μg/ml for 48 h. The shRNAs sequences of SENP3:

Sh-SENP3-1: 5′- CTATACAAGGGACCGGGTCC -3′.

Sh-SENP3-2: 5′- CCAGGCGGGAGCGTCTTCGT -3′.

### Co-IP assay

As we previously reported [23], total protein was collected using lysis buffer (#9803, Cell Signaling Technology, CST). A co-IP assay was conducted to investigate protein–protein interactions using a Dynabeads™ Kit (#14311D, Invitrogen). According to the kit’s specifications, specific antibodies were coupled with 1.5 mg of dynabeads for 16–24 h. Next, the antibody–dynabeads mixture were incubated with cell lysates for 1–2 h. The antibody–dynabeads–protein complex was washed three times with PBS-Tween-20 and then mixed with 1× blue loading buffer (#7722, Cell Signaling Technology). The mixture was heated in a metal bath at 70 ℃ for 5 min. The targeted proteins in supernatant were then collected by centrifugation at 13,000 rpm for 3 min.

In experiments to detect SUMOylation levels, it was also necessary to add freshly prepared 20 mM *N*-ethylmaleimide (NEM) to the lysis buffer, which was crucial to prevent further deSUMOylation of proteins, thereby enhancing the detection efficiency of SUMO conjugates[26–28]. Beyond this, all other steps were in accordance with the specifications.

### LC–MS/MS assay

For liquid chromatography tandem mass spectrometry (LC–MS/MS) analysis, we collected whole-cell lysates and performed Co-IP using the corresponding antibodies. We then proceeded with SDS–PAGE to separate the proteins, which were subsequently developed by silver staining (#P0017S, Beyotime). The gel was thoroughly rinsed with ddH_2_O and treated with a destaining reaction. The proteins were digested with trypsin into peptides, centrifugally concentrated, and dried. The peptides were resuspended in acetonitrile/formic acid, reconstituted in Nano-LC Solvent A (0.1% formic acid in water), and loaded onto a nanoViper C18 trap column for an Easy nLC 1200 separation system (ThermoFisher). After a 5-min desalting process with solvent A (water/acetonitrile/formic acid, 98/2/0.1%), a 60-min gradient elution was executed with solvent B (water/acetonitrile/formic acid, 2/98/0.1%) on an analytical column. The resulting peptides were analyzed using a ThermoFisher Q Exactive mass spectrometer with a Nano Flex source (ThermoFisher, USA). Data was acquired using an ion spray voltage of 1.9 kV and an interface heater temperature of 275 °C. The raw MS/MS data was processed with PEAKS Studio 8.5 for protein identification and quantification.

### Western blotting

Western blotting was used to detect protein levels. Total proteins were extracted from PCa cells and stored at −80 °C. Protein samples were separated by SDS–PAGE and transferred to PVDF membranes. Subsequently, the samples were incubated with 5% defatted milk for 1 h and then with appropriate primary antibodies at 4 °C overnight. Secondary horseradish peroxidase (HRP)-linked antibodies were then incubated with the samples at 24 °C for 1 h. Finally, the proteins were detected using either ECL reagent (#sc-2048, Santa Cruz Biotechnology) or enhanced ECL reagent (#FD8030, FDbio Science, Hangzhou, China), and then developed using X-ray films.

### Immunofluorescence assay

The immunofluorescence assay was used to investigate protein–protein interactions, as previously reported [29]. PCa cells were transfected with HA-SIX1 or FLAG-SENP3 plasmids for 48 h, fixed with 4% paraformaldehyde at room temperature for 15 min, and permeabilized with 0.5% Triton X-100 in PBS for 10 min. After blocking with 5% BSA at room temperature for 30 min and washing with PBS three times, the cells were incubated with primary antibodies at 4 °C overnight, followed by incubation with fluorescent secondary antibodies for 1 h. Finally, the cell nuclei were stained with DAPI (#ab104139, Abcam), and digital photographs were collected using a ZEISS confocal microscope (#LSM980, ZEISS).

### RNA extraction and quantitative real-time PCR analysis

Total RNAs extracted from PCa cells were reverse transcribed into cDNA as previously reported [30]. In simple terms, after the cells were washed with cold PBS, TRIzol was added for lysis. The mixture was incubated for 5 min and then transferred to 1.5 mL EP tubes. After centrifugation, the supernatant was taken, mixed with chloroform, and centrifuged again to obtain the aqueous phase. This was mixed with isopropanol and allowed to stand for 10 min. After centrifugation, the supernatant was discarded, and the pellet was washed successively with 75% and 95% ethanol, dried, and dissolved in DEPC water. The RNA purity was tested, and the 260/280 ratio should be between 1.9 and 2.0. The RNA was stored short-term at −20 °C, long-term at −80 °C, or reverse transcribed into cDNA.

Subsequently, quantitative real-time PCR analysis was performed using a TB Green® Premix Ex Taq™ II kit (#RR820A, TakaRa) with an ABI StepOnePlus™ Real-Time PCR System. All tests were independently repeated in triplicate. The PCR primers were as follows [7, 16]:

Forward-human SENP3: 5′‐CAAAGTCTCCTCTGGACCCTG‐3′;

Reverse-human SENP3: 5′‐TGCTGCACACATTGCTGATGAG‐3′.

Forward-human SIX1: 5′-AAGGAGAAGTCGAGGGGTGT-3′.

Reverse-human SIX1: 5′-TGCTTGTTGGAGGAGGAGTT-3′.

Forward-human GAPDH: 5′‐GGTATCGTGGAAGGACTCATGAC‐3′.

Reverse-human GAPDH: 5′‐ATGCCAGTGAGCTTCCCGTTCAG‐3’.

### Cell proliferation assay

Cell proliferation consisted of cell viability determination, EdU staining analysis (#C10310-1, Ribobio), and live cell assay. We performed the cell viability assay using the MTS kit (#G3581, Promega). The EdU staining analysis was conducted using a microscope (#BX53, OLYMPUS). For clonogenic assays, transfected PCa cells were re-plated in six-well plates and allowed to grow for at least 10 days. The cells were then stained with crystal violet (#C0121, Beyotime Biotechnology). All tests were repeated independently in triplicate. The live cell assay utilized an Incucyte® S3 Live Cell Analysis System (Sartorius, Goettingen, Germany). In short, a certain density of PCa cells (22Rv1: 1500; PC3: 1000) were plated per well in 96-well plates. After 1 day, the cells were transfected with shRNAs. Cell confluence values were measured every 6 h for 6 days and then used to construct proliferation curves.

### Scratch and cell migration assay

Scratch assays were performed using 25 culture inserts, with three wells for self-insertion (#80,369, ibidi, Gräfelfing, Germany). After 48 h of siRNAs transfection, PCa cell lines in each treatment group were resuspended in new culture medium and seeded onto six-well plates with inserts. When cell confluence reached 90%, the inserts were removed, and 2 ml of the corresponding medium was added to each well. Scratch distances of PCa cells at 0, 12, and 72 h were observed and recorded.

Cell migration assay was performed using 24-well transwell chamber (#3422, Corning, New York, USA). As previously reported [25], 5 × 10^4^-treated 22Rv1 or 8 × 10^3^ PC3 were resuspended with 200 μl serum-free medium and plated into the upper side of the chamber; the lower side chamber was filled with 600 μl of the corresponding medium supplemented with 10% FBS. After incubation for an appropriate time, 4% paraformaldehyde was used to fix the cells on the underside for 15 min, and then the migrating cells adhering to the underside were stained with crystal violet for 15 min. Digital photographs were collected using a microscope (#BX53, OLYMPUS).

### Immunohistochemistry assay

The expression of SENP3 in patients from the Affiliated Cancer Hospital & Institute of Guangzhou Medical University (Guangdong, China) was detected by immunohistochemistry. As we previously reported [23], the immobilized samples were incubated according to the specifications of the MaxVision™ HRP-Polymer kit (#KIT-5004, Maixin Biol, Fujian, China), then stained with DAB and counterstained with hematoxylin. Finally, the ImageJ software was used to analyze the digital images.

### 22Rv1 xenograft models

NOD-SCID mice were purchased from Charles River Laboratory (Beijing, China). As previously reported [23], the mice were randomly divided into three groups and housed in the Laboratory Animal Center of Guangzhou Medical University. Then, 2 × 10^6^ cells with stable expression of control shRNA, SENP3 shRNA-1, or shRNA-2 were injected subcutaneously into each mouse. The weight and tumor size of the mice were measured at specified times after inoculation. On the 16th day after tumor formation, all mice were euthanized by cervical vertebrae dislocation following CO_2_ asphyxiation. The tumor weights were then measured.

### Statistical analysis

One-way ANOVA or Student’s *t*-tests were used to analyze three or more independent datasets for statistical significance on GraphPad Prism 8 and SPSS 22.0. A *P* value of less than 0.05 (two-sided) was considered statistically significant.

## Results

### SENP3 promotes PCa proliferation via boosting cell cycle transition

**Fig. 1:**
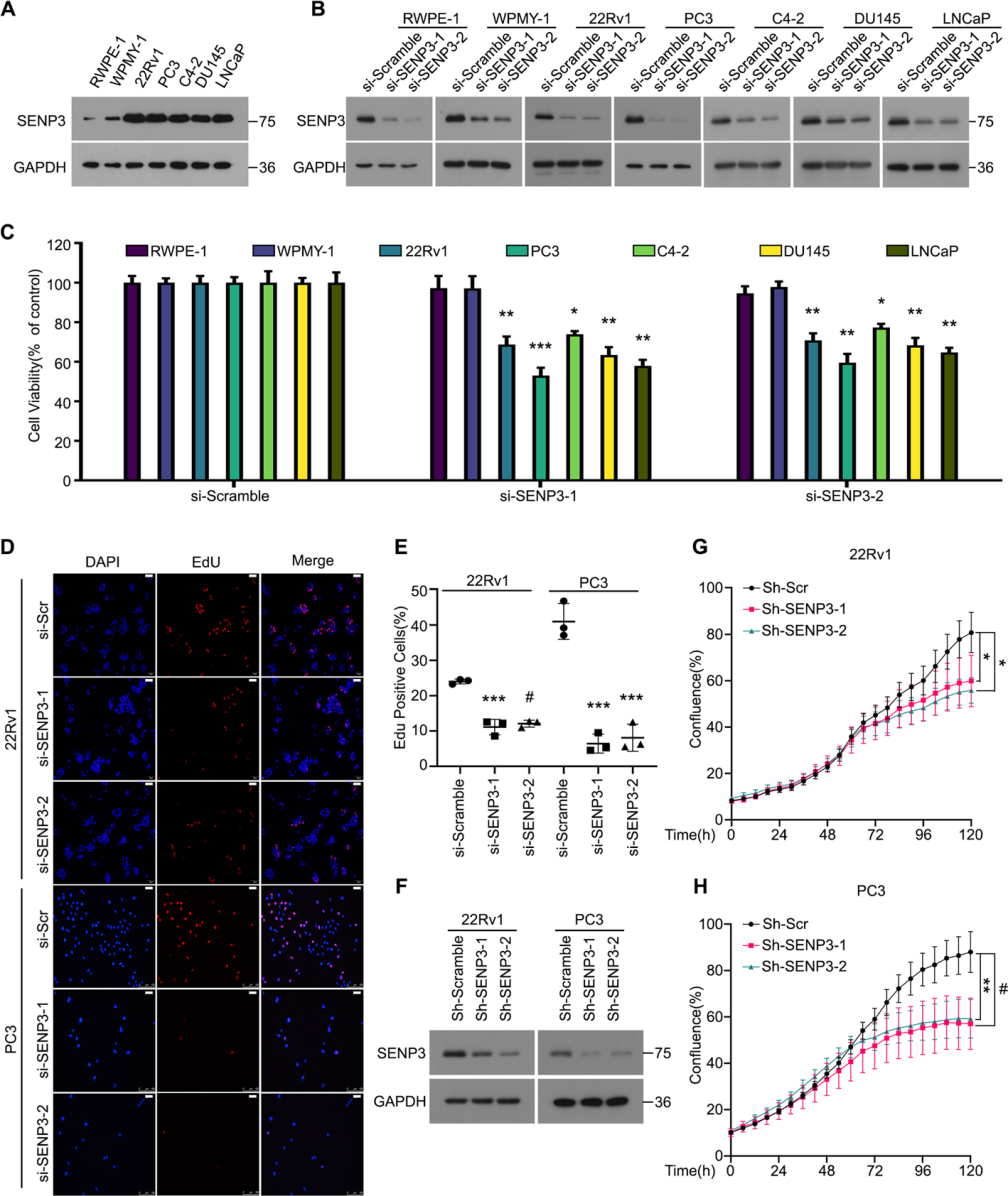
SENP3 promotes the proliferation of PCa. **A** The protein expression of SENP3 in the following cell lines: the normal human prostate cell lines (RWPE-1 and WPMY-1) and PCa cell lines (22Rv1, PC3, C4-2, DU145 and LNCaP). **B** The normal human prostate cell lines (RWPE-1 and WPMY-1) and PCa cell lines (22Rv1, PC3, C4-2, DU145 and LNCaP) were treated with SENP3 siRNAs or control siRNAs. The expression levels of SENP3 were determined by western blot. GAPDH was used as a loading control. **C** The indicated PCa cell lines were treated with SENP3 siRNAs or control siRNAs for 48 h. Cell viability was determined by MTS assay from three independent repeats. **D** and **E** 22Rv1 and PC3 cells were treated with SENP3 siRNAs for 48 h. DNA duplication was determined by EdU staining assay and representations are shown (Scale bars, 50 μm). **F** The expression levels of SENP3 were determined in the indicated PCa cells stably expressing SENP3 ShRNAs or control ShRNAs by western blot. GAPDH was used as a loading control. **G** and** H** PCa cell lines transfected with SENP3 ShRNAs for 24 h were subjected to 6 days confluence assay using an Incucyte®S3 Live Cell Analysis System. Mean ± SD (*n* = 3); *P* values were calculated using one-way ANOVA with Tukey test for multiple comparisons. *P < 0.05, **P < 0.01, ***P < 0.001, #*P* < 0.0001

**Fig. 2:**
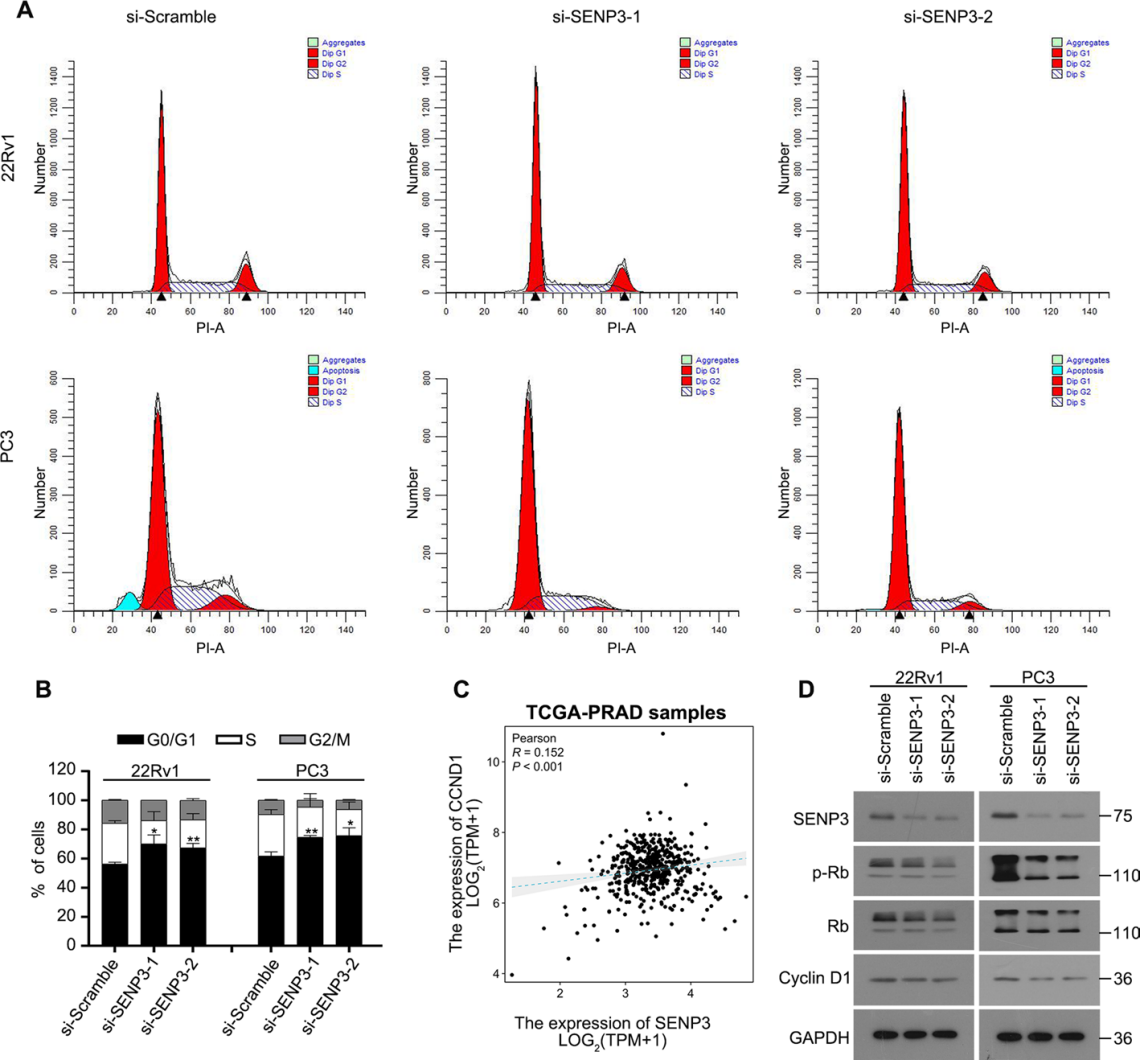
Inhibition of SENP3 arrested cell cycle of PCa. **A** and **B** PCa cells transfected with SENP3 siRNAs for 48 h were subjected to fluorescence-activated cell sorting analysis for cell cycle distributions. **C** The correlation between SENP3 and CCND1 (cyclin D1) expression in the TCGA-PRAD (prostate cancer) project from the TCGA database, utilizing non-duplicate clinical tumor samples. **D** Western blot assay was performed to test expression of SENP3, p-Rb, Rb, cyclin D1, and GAPDH. *P < 0.05, **P < 0.01, ***P < 0.001, #*P* < 0.0001

SENP3 is adept at detaching SUMO2/3 from substrates, with studies revealing its elevated expression in various pathologies and tumors [19, 21]. Yet, the role of SENP3 in PCa remains to be definitively characterized. To investigate the role of SENP3 in PCa, we first detected the expression of SENP3 in PCa lines and found that SENP3 was highly expressed in PCa (Fig. 1A). Subsequently, cell viability assays were used to measure the proliferation of normal prostate cell lines and PCa cell lines after treatment with SENP3-siRNAs. As expected, the knockdown of SENP3 decreased the viability of various PCa cell lines, but not human prostate stromal cell lines (Fig. 1B, C). Additionally, we confirmed that downregulation of SENP3 inhibited DNA replication in 22Rv1 and PC3 cells through EdU staining assay (Fig. 1D, E). To clarify the role of SENP3 depletion in PCa, we constructed stable SENP3-deficient 22Rv1 and PC3 cells using two independent shRNAs (Fig. 1F). Next, we evaluated their proliferation rates by the live-cell analysis system and observed that tumor cell lines require SENP3 for sustained proliferation (Fig. 1G, H). Through further flow cytometry analysis, we found that cell numbers were increased in the G0/G1 phase, indicating that downregulation of SENP3 inhibits the transition of PCa cells from G1 to S phase (Fig. 2A, B). We investigated the correlation between the mRNA expression levels of SENP3 and cyclin D1 in the TCGA database and observed a positive correlation between them (Fig. 2C). Western blot analysis further indicated that downregulation of SENP3 decreased the protein levels of cyclin D1, Rb, and p-Rb (Fig. 2D). These results suggest a pivotal role for SENP3 in cell cycle regulation, indicating that SENP3 facilitates the transition from the G1 phase to the S phase to promote PCa progression.

### SENP3 DRIVES migration OF PCa Cells

**Fig. 3:**
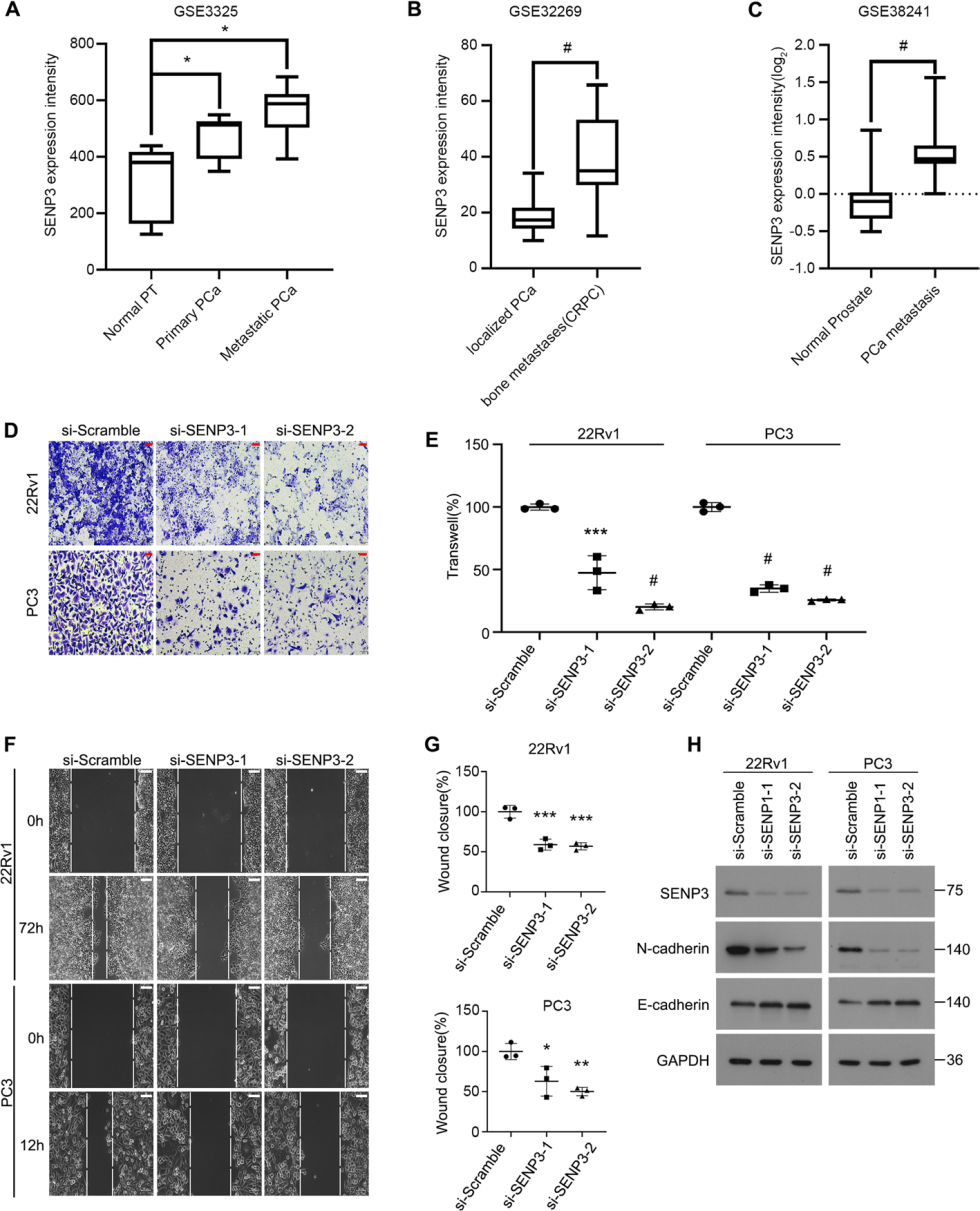
Inhibition of SENP3 impeded PCa migration. **A**–**C** The SENP3 mRNA levels were analyzed on the basis of three different GSE datasets. Unit: MAS5-calculated signal intensity (GSE3325), MAS5.0 signal intensity (GSE32269), and normalized log_2_ ratio (Cy5/Cy3) representing test/reference (GSE38241). PT, prostate tissue; PCa, prostate cancer. **D** and **E** Transwell migration assay was performed in 22Rv1 and PC3 cells transfected with SENP3 siRNAs or control for 24 h. Representations are shown (scale bars, 50 μm). **F** and **G** Scratch assay was performed in 22Rv1 and PC3 cells transfected with SENP3 siRNAs or control for 12 h or 72 h. Representations are shown (scale bars, 50 μm). **H** Western blot assay was performed to test expression of SENP3, N-cadherin, E-cadherin, GAPDH. *P < 0.05, **P < 0.01, ***P < 0.001, #*P* < 0.0001

Tumor metastasis is a challenging problem that greatly worsens prognosis of patients. To investigate the involvement of SENP3 in PCa metastasis, we first analyzed several publicly available Gene Expression Omnibus (GEO) databases [31–33] and observed that the SENP3 mRNA levels were significantly increased in metastatic PCa tissues, compared with the normal prostate or primary PCa tissues (Fig. 3A–C). By using transwell and scratch experiments, we next observed that the migration rate was significantly reduced in PCa cells transfected with SENP3 siRNAs (Fig. 3D–G). Furthermore, the expression of migration-related proteins was altered post the silence of SENP3, with downregulation of N-cadherin and upregulation of E-cadherin (Fig. 3H). These results demonstrate that SENP3 plays a critical role in boosting the proliferation and metastasis of PCa.

### SIX1 interacts with the SUMO-specific protease SENP3

**Fig. 4:**
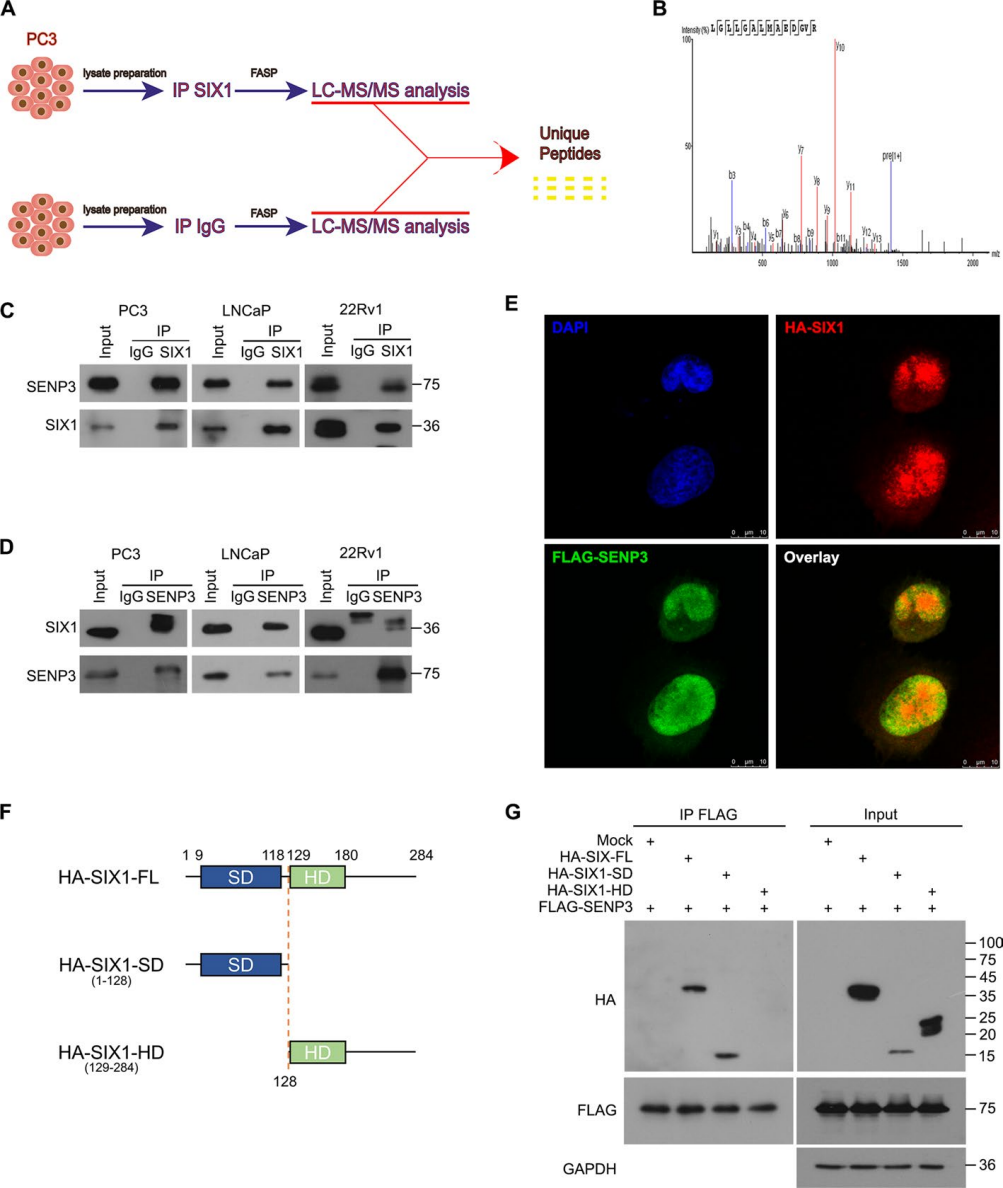
SENP3 interacts with SIX1 in PCa cells. **A** Schematic diagram of endogenous SIX1 and IgG immunoprecipitation combined with mass spectrometry on PC3. **B** Ambipolar ion peaks of SENP3 are shown. **C** Co-IP assay was performed using SIX1 antibody and control IgG antibody, which subjected to immunoblot for SENP3 and SIX1. **D** CO-IP assay was performed using SENP3 antibody and control IgG antibody, which subjected to immunoblot for SENP3 and SIX1. **E** Immunofluorescence assay was performed using FLAG-tag and HA-tag antibodies in the indicated 22Rv1 cells transfected with FLAG-SENP3 and HA-SIX1 plasmids (scale bar, 10μm). **F** Full-length (FL) and truncated mutants of SIX1 fused with HA-tag were engineered and co-transfected with FLAG-SENP3 into HEK293T cells for 48 h. **G** Co-IP assay was performed using FLAG-tag antibody beads and immunoblotted for FLAG, HA

Us and another group have shown that SIX1, a transcriptional factor that mediates embryo development, is reactivated and promotes the progression of various cancers, including PCa, breast cancer and hepatocellular carcinoma, etc. [6, 10, 34, 35]. However, whether SUMOylation regulates SIX1 function or PCa development remains unclear. By using SIX1-based immunoprecipitation coupled with unbiased mass spectrometry, we identified SENP3 as a potential player that interacts with SIX1 (Fig. 4A, B). To confirm the interaction, we further validated the interaction of SENP3 with SIX1 by co-immunoprecipitation (co-IP) of endogenous SENP3 or SIX1 in 22Rv1, LNCaP, and PC3 cells (Fig. 4C, D). In addition, the SENP3–SIX1 interaction occurred primarily in the nucleus of PCa cell lines instead of the cytoplasm (Fig. 4E). SIX1 consists of a SIX domain (SD) for protein interactions and a homeodomain (HD) for DNA binding [5]. To further observe the structural binding region of SIX1, two truncated mutants (SD and HD) of SIX1 and FLAG-SENP3 were transfected into HEK293T cells (Fig. 4F, G). We observed that full length HA-SIX1 and SD were able to interact with SENP3, indicating that the N-terminal (1–128) of SIX1 is required for the binding of SENP3.Taken together, these results suggest that SENP3 can interact with the SD domain of SIX1 in the nucleus of PCa cells.

### SENP3 prevents SIX1 degradation by deSUMOylation activity

**Fig. 5:**
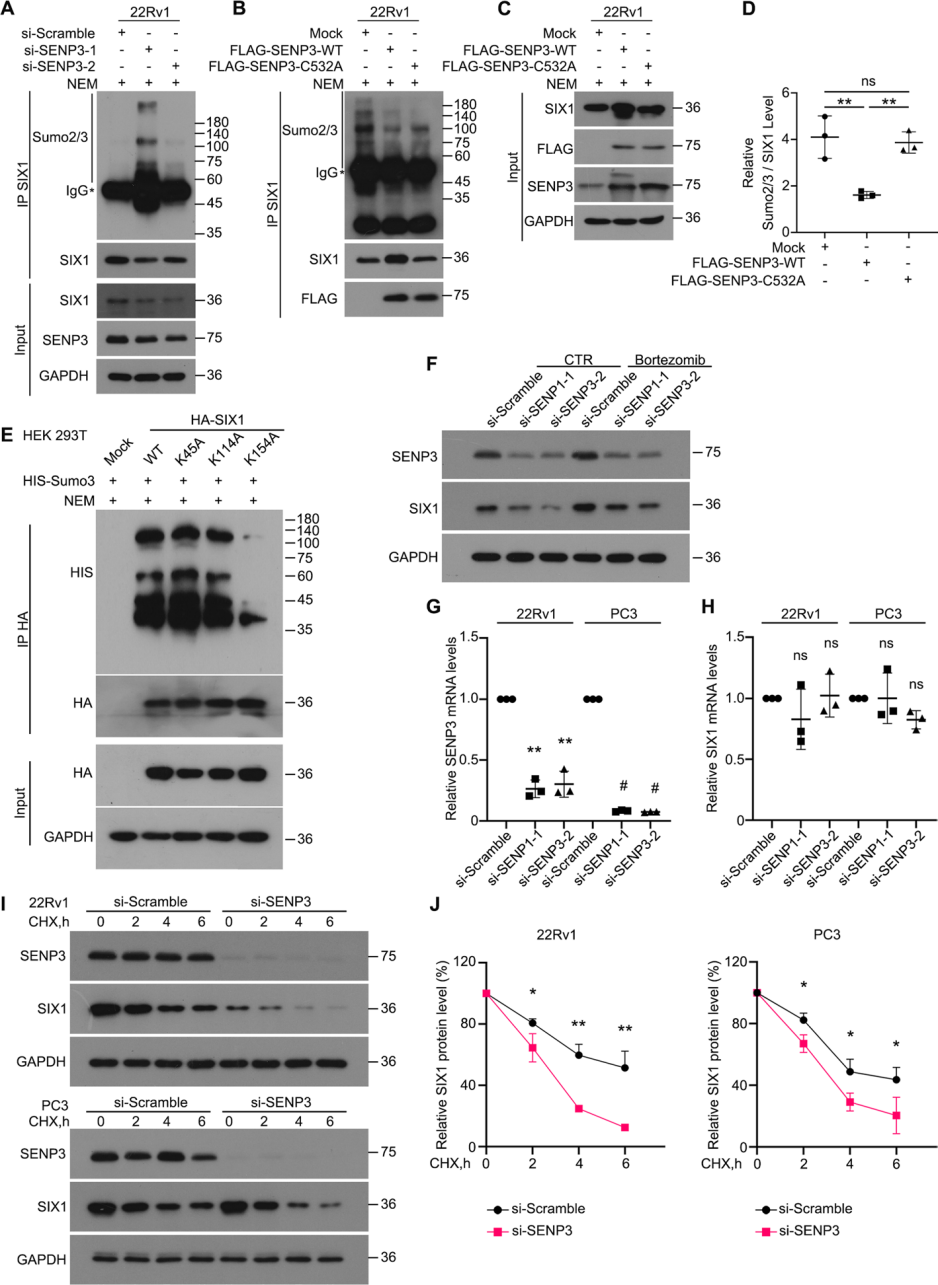
SENP3 mediates the deSUMOylation and stability of SIX1. **A** Co-IP assay was performed using SIX1 antibody and immunoblotted for SUMO2/3 and SIX1 in 22Rv1 treated with SENP3 siRNAs for 42 h and exposed to NEM (20 mM) for 6 h before harvest. **B-D** Co-IP assay was performed using SIX1 antibody and immunoblotted for SUMO2/3 and SIX1 in 22Rv1 transfected with SENP3-relative plasmid for 42 h and exposed to NEM (20 mM) for 6 h before harvest. **E** Co-IP assays were performed using HA-tag antibodies in lysates from HEK293T cells transfected with various Lys-mutant types of HA-SIX1 and His-SUMO3 plasmids for 48 h, subjected to the immunoblotting for His-tag and HA-tag. NEM was used to treat the cells for 6 h before harvest. **F** Immunoblot analysis of SIX1 in PC3 cells treated with SENP3 siRNAs for 36 h with or without Bortezomib (BTZ) for 12 h. **G** and** H** RT–PCR assay for SENP3 and SIX1 was performed in 22Rv1 and PC3 treated with SENP3 siRNAs for 12 h. **I** and **J** 22Rv1 and PC3 cells were exposed to cycloheximide (CHX, 50 μg/ml) for indicated time with or without the pre-treatment of siSENP3. The expression level of SIX1 was determined by immunoblot. Mean ± SD (*n* = 3) *P < 0.05, **P < 0.01, ***P < 0.001, #*P* < 0.0001

We have confirmed the interaction between SENP3 and SIX1. Next, we analyzed the significance of the SIX1-SENP3 interaction on SIX1 expression and function. Recognizing SENP3’s role as a key protease in removing SUMO2/3 from modified proteins, we utilized two independent siRNA of SENP3. Through co-IP and western blot analysis, we investigated the presence of SUMO2/3-linked SUMOylation of SIX1, also confirmed that SENP3 depletion led to an upregulation of endogenous SUMOylation of SIX1 (Fig. 5A and Additional file 1: Fig. S1). To further demonstrate that the deSUMOylation activity of SENP3 is necessary for the regulation of SIX1 SUMOylation levels, we designed wild-type SENP3 (SENP3-WT) and an inactive mutant (SENP3-C532A) [28]. 22Rv1 cells were transfected with both, and then co-IP and immunoblotting analysis revealed that the wild-type SENP3, but not the SENP3-C532A mutant, downregulated the SUMOylation levels of SIX1 in 22Rv1 (Fig. 5B–D).

Most SUMO-modified proteins contain the tetrapeptide motif Ψ-K-x-D/E (where Ψ is an hydrophobic residue, K is Lys residue conjugated to SUMO, x is any amino acid, D or E is an acidic residue) [11]. To identify the SUMOylation site on SIX1, we used the public tool, SUMOplot (http://www.abgent.com/doc/sumoplot) to predict the potential modification sites of SIX1 and then constructed Lys-mutant plasmids. These plasmids were transfected into HEK293T cells, respectively. Our co-IP results showed that the SUMOylation level of SIX1 (K154A), but not other mutant type of SIX1, was downregulated, indicating that K154 is a critical SUMOylation site on SIX1 (Fig. 5E). Given that knockdown of SENP3 reduced the protein expression levels of SIX1, while overexpression of SENP3-WT increased the protein them, we wondered if bortezomib (BTZ), a proteasome inhibitor, could reverse the downregulation of SIX1 induced by SENP3 knockdown. As expected, the immunoblotting analysis revealed that BTZ rescued the downregulation of SIX1 caused by SENP3 ablation (Fig. 5F). In addition, we found that the knockdown of SENP3 did not affect SIX1 mRNA levels (Fig. 5G, H). To further explore whether SENP3 can affect the stability of SIX1, a cycloheximide (CHX) chase analysis was performed in SENP3-knockdown PCa cell lines. Targeting SENP3 with siRNA noticeably decreased the half-time period of SIX1 (Fig. 5I, J), indicating that SENP3 may contribute to the protein stability of SIX1.Taken together, these results suggest that SENP3 deSUMOylates and stabilizes SIX1.

### SENP3-driven PCa cell proliferation and metastasis depends on SIX1

**Fig. 6:**
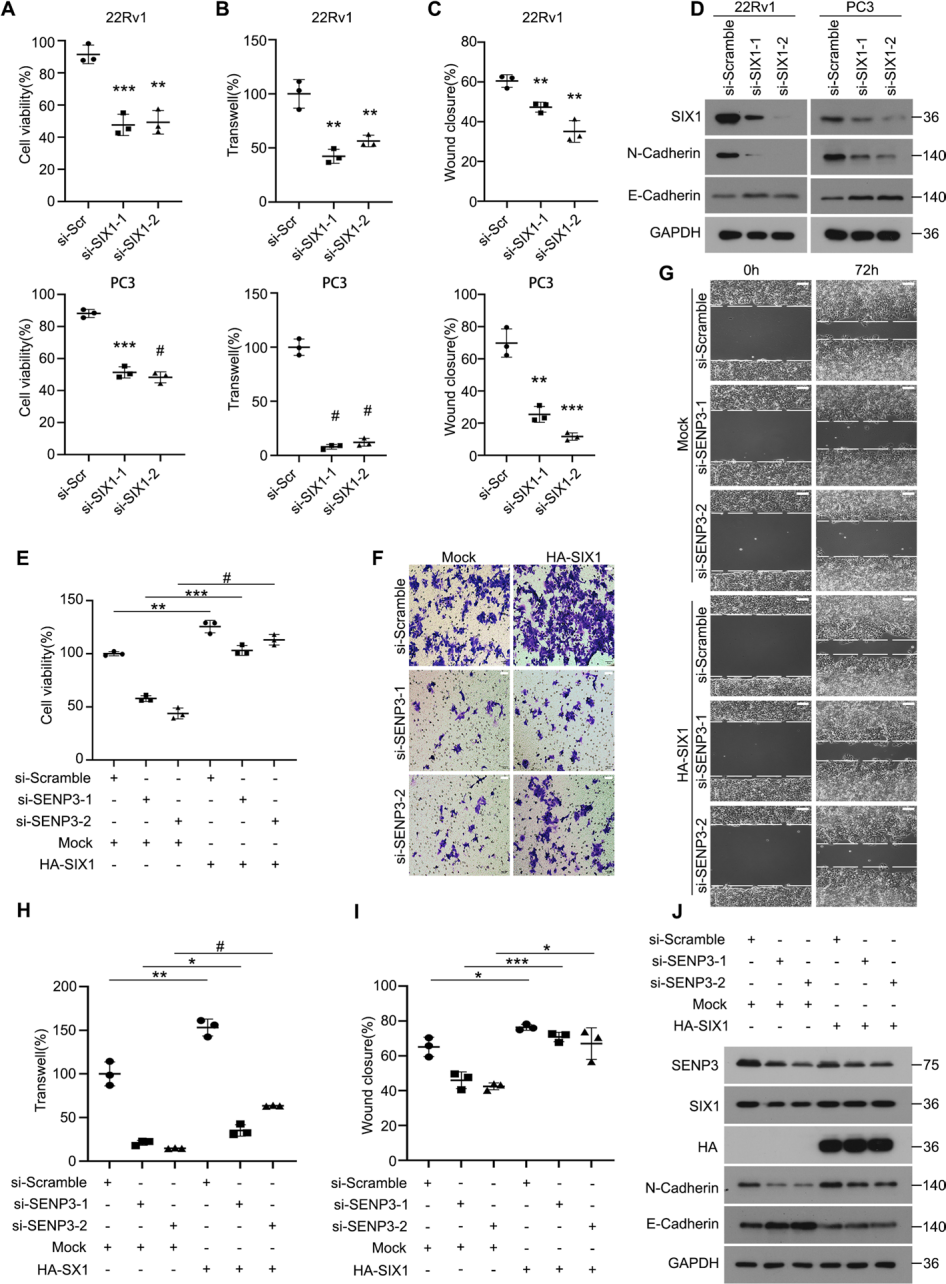
SENP3-driven PCa cell proliferation and metastasis depends on SIX1. **A**–**C** The PCa cells 22Rv1 and PC3 were transfected with SIX1 siRNAs or control siRNAs for 48 h, and subjected to MTS assay, transwell migration assay, and scratch assay. **D** Western blot assay was performed to test expression of SIX1, N-cadherin, E-cadherin, and GAPDH.** E**–**J** 22Rv1 cells were transfected with SENP3 siRNAs for 24 h and then transfected with or without the transfection of HA-SIX1 for 48 h. **E** Cell viability was determined by MTS assay. **F** and **H** Transwell migration assay representations are shown. **G** and **I** Scratch assay representations are shown (scale bars, 50 μm). **J** Western blot assay was performed to test expression of SENP3, SIX1, HA-tag, N-cadherin, E-cadherin, GAPDH. *P < 0.05, **P < 0.01, ***P < 0.001, #*P* < 0.0001

Next, we aimed to determine whether SENP3-regulated SIX1 is required for cell proliferation and migration in PCa cell lines. As expected, cell viability, transwell assays, and scratch assays revealed that SIX1 deletion significantly impaired the proliferation and migration of PCa cell lines (Fig. 6A–C and Additional file 1: Fig. S2A, B). Accordingly, immunoblot analysis showed that SIX1 deletion also significantly altered the expression of migration-related proteins (Fig. 6D). These findings suggest a potential role for SIX1 in promoting PCa proliferation and migration.

To further confirm that SIX1 is the downstream target of SENP3 function, we overexpressed HA-SIX1 among PCa cell lines treated with SENP3 siRNA. Overexpression of HA-SIX1 in the cells partially restored the SENP3 knockdown-induced changes in cell viability, transwell assays and scratch assays, and E-cadherin and N-cadherin protein expression (Fig. 6E–J). Taken together, these findings suggest that SENP3-mediated protein stabilization of SIX1 contributes to PCa cell proliferation and migration.

### SENP3 ablation suppresses the PCa growth in vivo

**Fig. 7:**
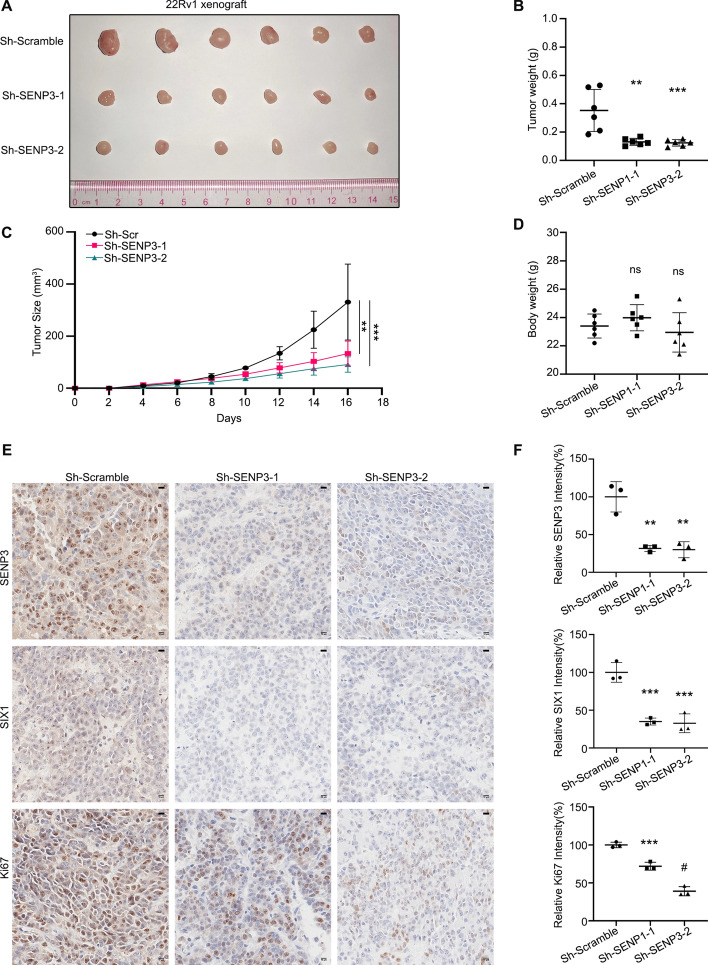
Knockdown of SENP3 suppresses the PCa xenografts. **A** 22Rv1 xenografts stably transduced with SENP3 ShRNA or the control ShRNA. *n* = 6 pre-group. **B** and **C** Tumor weight growth curves were recorded. **D** Body weight of mice with xenografts. **E** Expression of SENP3, SIX1, and Ki67 were detected in indicated xenograft by immunohistochemistry assay (scale bar, 10 μm). **F** Quantification of SENP3, SIX1, and Ki67 are shown. *P < 0.05, **P < 0.01, ***P < 0.001, #*P* < 0.0001

Next, we established 22Rv1 xenografts on NOD-SCID mice by subcutaneously injecting 22Rv1 cells stably expressing SENP3 shRNAs or the control shRNAs. Eventually, the SENP3 ablation xenografts had a significantly diminished tumor weight and size (Fig. 7A–C) without influencing the weight of various groups (Fig. 7D). Furthermore, immunohistochemistry assays were performed to detect the expression of SENP3, SIX1, and Ki67, among which the control group was significantly higher than the others (Fig. 7E, F). Animal experiments further verified that SENP3 promotes progression of PCa in vivo.

### Clinical relevance of SENP3 in PCa

**Fig. 8:**
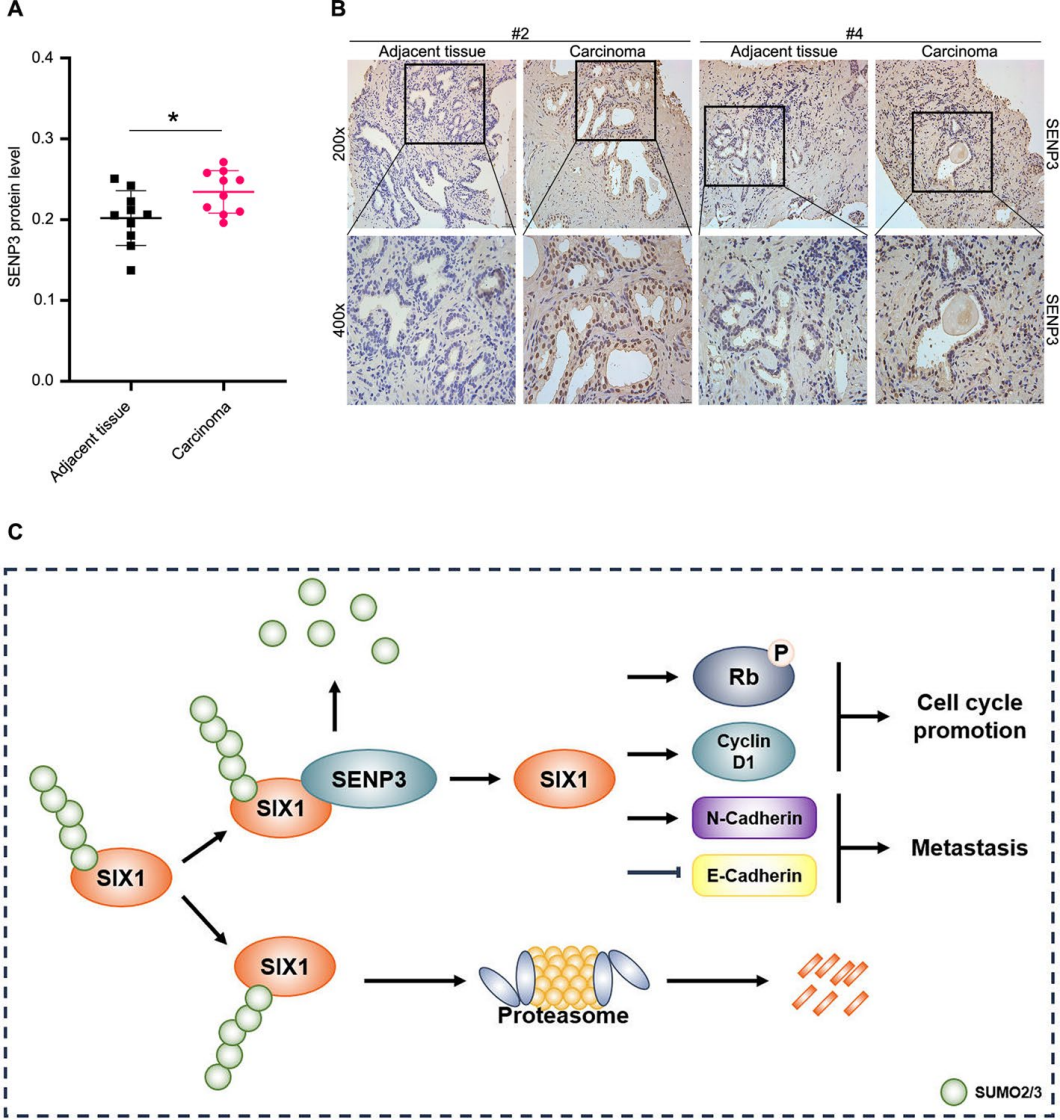
Clinical relationship of SENP3. **A** Immunohistochemistry assay was performed in PCa and normal adjacent tissue (*n* = 10); *P* values were calculated using Paired t test for comparisons. **B** Representations of SENP3 are shown. **C** Graphical summary of SENP3 regulated proliferation and metastasis of PCa. *P < 0.05

Finally, to investigate the clinical relevance of SENP3 in PCa, we analyzed SENP3 expression in ten pairs of PCa and adjacent normal tissues derived from patients by immunohistochemistry, and found that SENP3 was upregulated in PCa (Fig. 8A, B). This evidence supports our preclinical findings that SENP3 contributes to PCa growth, suggesting that SENP3 has potential as a therapeutic target.

In conclusion, we revealed that the SENP3–SIX1 axis promotes the growth and metastasis of PCa cells (Fig. 8C). Targeting SENP3-mediated SIX1 stability may offer a potentially new strategy for metastatic PCa.

## Discussion

SUMO modification is a dynamic and reversible process that plays an indispensable role in fine-tuning cancer functions and pathological processes [11]. It has been reported that SUMOylation plays a critical role in the progression of PCa. Research on PCa has revealed that sustained SUMOylation of ATF3 can activate the cell cycle and promote tumor proliferation [36], while SUMOylated flotillin-1 can undergo nuclear translocation to promote epithelial–mesenchymal transition (EMT) transformation [37]. Accumulating evidence suggests that changes in SUMOylation also play a critical role in immunosuppression and angiogenesis in PCa [38, 39]. Thus, incorporating drugs targeting molecules in the SUMO pathway into the treatment of PCa may potentially bring greater benefits to patients.

Our previous study confirmed that SIX1 is an oncogene in PCa [10]. However, whether SIX1 can undergo SUMOylation has not been reported. In this study, we discovered that SENP3 plays a key role in the proliferation and metastasis of PCa cells. SENP3, a member of the SENPs family, mediates the removal of substrates in SUMOylation, primarily targeting the removal of SUMO2/3 [28]. Elevated levels of SENP3 have been observed in various diseases and tumors, participating in physiological or pathological processes such as vascular remodeling, autophagy, drug resistance, and immune evasion by modulating the functions or stability of different target molecules [19–22, 40].

Some studies have shown that SUMO modification can promote the formation of ubiquitin chains on target proteins, promoting their degradation [41–43]. In this study, we further identified that the stability of SIX1 is regulated by the deSUMOylation activity of SENP3. Knockdown of SENP3 resulted in increased SUMOylation levels of SIX1, leading to a decrease in the protein levels of SIX1. Consequently, Bortezomib (BTZ) can partially reverse the downregulation of SIX1 levels caused by SENP3 ablation, indicating that SUMOylation of SIX1 may recruit ubiquitin, leading to degradation through the proteasome system. In summary, SENP3 regulates SIX1 through deSUMOylation, stabilizes its protein levels, and thereby exerts oncogenic activity.

Proliferation and metastasis contribute to the further deterioration of many cancers [4]. On one hand, uncontrolled cell proliferation is a necessary mechanism for the rapid growth of tumors, and cell cycle arrest can significantly reduce the proliferation rate of tumors [7, 39, 44]. By knocking down SENP3, we halted the transition of PCa cells from the G1 phase to the S phase. The downregulation of the key protein cyclin D1, which is consistent with the regulatory role of SIX1 in Cyclin D1 [44], played a crucial role in this process. On the other hand, the major transition in expression from E-cadherin to N-cadherin in epithelial cells is a hallmark of the progression of epithelial–mesenchymal transition (EMT), which is associated with the progression of various cancers [44]. Previous studies have demonstrated the involvement of SENP3 in EMT [45, 46]. In line with this, our research reveals that SENP3 can promote the transition of EMT in the metastatic process of prostate cancer. The results indicate that SENP3 reduces the expression of E-cadherin in PCa cells, increases the expression of N-cadherin, thereby facilitating the invasion and dissemination of cancer cells. This suggests that as prostate cancer progresses to advanced stages, SENP3 may play a critical role in driving the invasiveness of prostate cancer.

In recent years, technologies such as single-cell sequencing of cancer cells and organoid cultures have enabled the real-time and specific acquisition of patient information, guiding targeted clinical treatments [47–50]. This underscores the importance of identifying different oncogenic molecules in different tumors. Simultaneously, the search for and development of molecular targeted therapies has become increasingly crucial. Although our study found that SENP3 is highly expressed in clinical patients and exerts oncogenic activity by regulating SIX1 through deSUMOylation, several limitations of this study are worth discussing. First, our findings were mostly obtained from knockdown assays, but lack the support of knockout assays. Second, we did not establish PCa xenografts in situ in the animal experiments, thus the role of SENP3 in tumor microenvironment remains to be further explored. Furthermore, this study only obtained a small number of clinical samples, further validation in multicenter, different patient cohorts is essential to determine translational value of SENP3. We will continue to explore additional roles and mechanisms of SUMOylation in PCa and seek suitable intervention drugs, aiming to incorporate different targets into genetic references for personalized treatment. Our research findings suggest the involvement of the SENP3-SIX1 axis in promoting the growth and metastasis of PCa, providing a reliable basis for considering SENP3 as a therapeutic target.

## Conclusions

Our study revealed that SENP3 facilitates the development and metastasis of PCa by mediating the deSUMOylation of SIX1. These results provide significant insights into the molecular mechanisms underlying metastatic lesions in PCa and enhance our understanding of SUMOylation, potentially paving the way for novel therapeutic approaches.

## Supplementary Information

Additional file 1.

## Data Availability

For the analysis of SENP3 expression, this study employed data from three Gene Expression Omnibus database datasets: GSE3325, GSE32269, and GSE38241. Comprehensive supporting data related to this research can be found within the main article and its supplementary materials. These data are available from the corresponding authors upon a reasonable request.

## Abbreviations

PCa: Prostate cancer
SENP3: Sentrin/SUMO-specific protease 3
SIX1: Sine oculis homeobox homolog 1
SUMO: Small ubiquitin-like modifier
PTM: Post-translational modification
Co-IP: Co-immunoprecipitation
mRNA: Messenger RNA
qPCR: Quantitative real-time PCR
GSE: Genomic spatial event
NEM: N-Ethylmaleimide
BTZ: Bortezomib
CHX: Cycloheximide
EMT: Epithelial–mesenchymal transition
AR: Androgen receptor
USP1: Ubiquitin-specific peptidase 1
GRP75: Glucose regulatory protein 75
IHC: Immunohistochemistry
