# Supplementary material for: SENP3 mediates deSUMOylation of SIX1 to promote prostate cancer proliferation and migration
Source: Cell Mol Biol Lett. 2024 Dec 2;29:146. doi: 10.1186/s11658-024-00665-8 (PMC11613746; doi:10.1186/s11658-024-00665-8)
Supplement: Supplementary file 1 — Additional file 1. [file 11658_2024_665_MOESM1_ESM.docx]

**SENP3 mediates deSUMOylation of SIX1 to promote prostate cancer proliferation and migration**

Zhenlong Shao^1,†^, Shutong Liu^1,†^, Wenshuang Sun^1,†^, Xuefen Zhuang^1^, Shusha Yin^1^, Ji Cheng^1^, Xiaohong Xia^1^, Yuning Liao^1,*^, Jinbao Liu^1,*^, Hongbiao Huang^1,*^


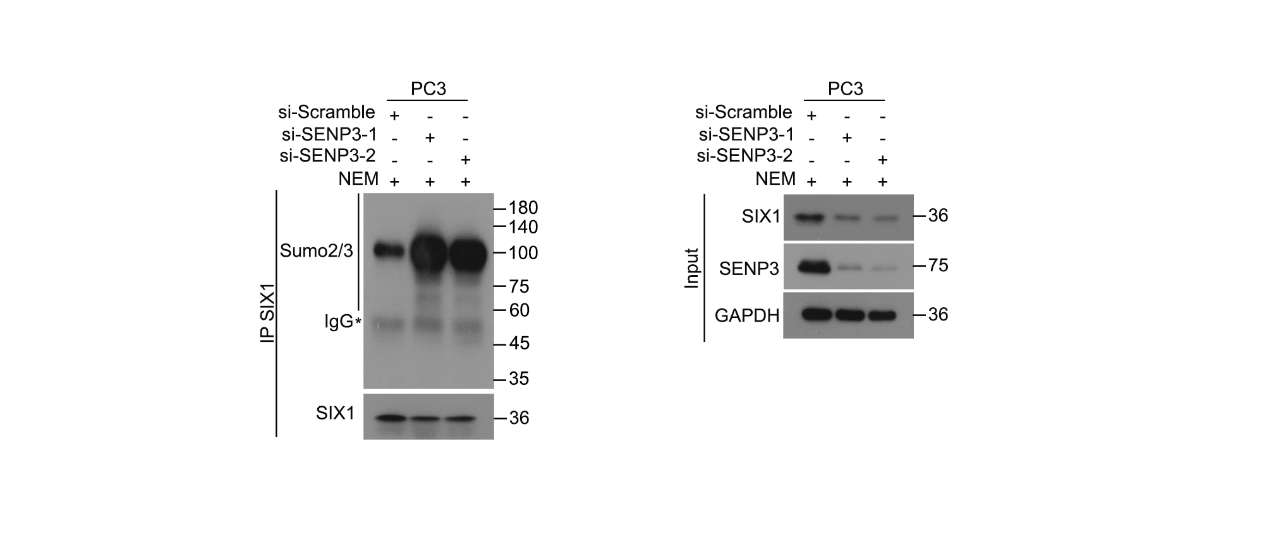


**Fig S1.** Knockdown of SENP3 increases the SUMOylation and reduce stability of SIX1. PC3 cells were transfected with SENP3 SiRNAs for 48 hours and exposed to NEM (20 μM) for 6 h before harvest and subject to Co-IP assay was performed using SIX1 antibody and immunoblotted for SUMO2/3 and SIX1. SA00001-7L (Proteintech, Manchester, UK) as secondary antibody in a dilution at 1: 5000.


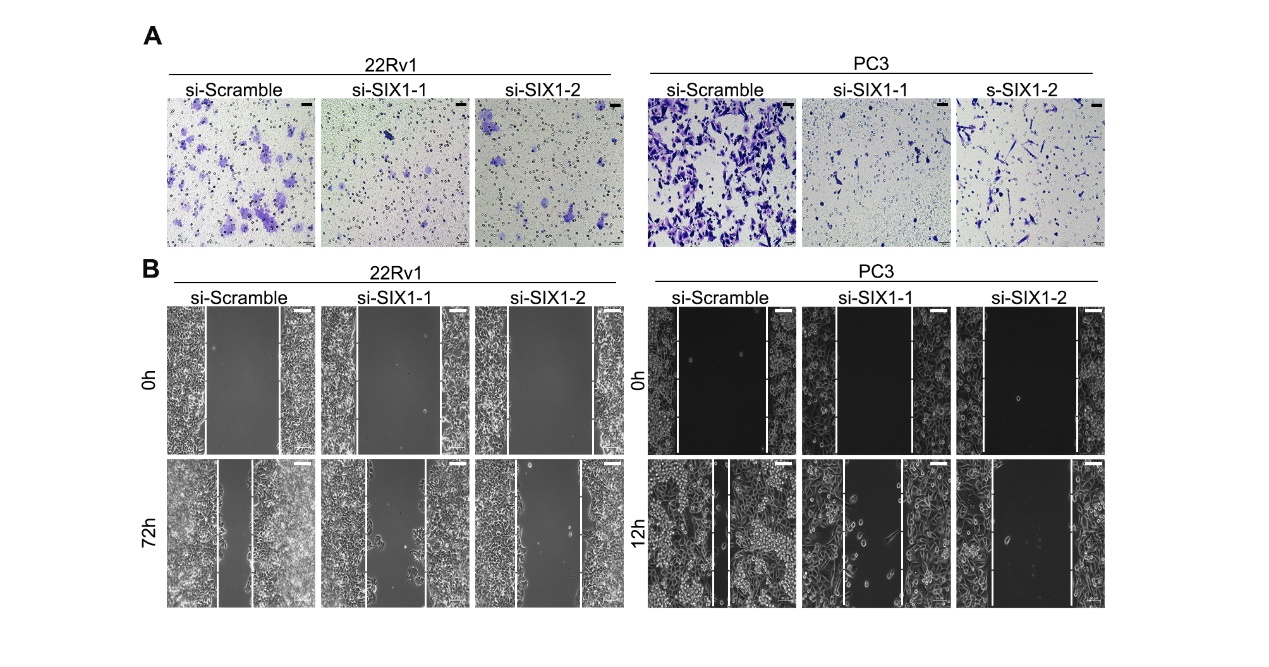


**Fig S2.** Inhibition of SIX1 impeded PCa migration. The PCa cells 22Rv1 and PC3 were transfected with SIX1 siRNAs or control siRNAs for 48 hours, and subjected to MTS assay, Transwell migration assay and Scratch assay. **A** Transwell migration assay representations are shown. **B** Scratch assay representations are shown (Scale bars, 50 μm).
